# Quantitative Analysis of Nucleic Acid Hybridization on Magnetic Particles and Quantum Dot-Based Probes

**DOI:** 10.3390/s90705590

**Published:** 2009-07-14

**Authors:** Sun Hee Lim, Felix Bestvater, Philippe Buchy, Sek Mardy, Alexey Dan Chin Yu

**Affiliations:** 1 Diagnostics, Institut Pasteur Korea, 696 Sampyeong-dong, Bundang-gu, Seongnam City, Gyeonggi-do, Korea, 443-270; 2 Cell Biophysics, Institut Pasteur Korea, 696 Sampyeong-dong, Bundang-gu, Seongnam City, Gyeonggi-do, Korea, 443-270; E-Mail: mailto:f.bestvater@ip-korea.org; 3 Virology Unit, Institut Pasteur in Cambodia, 5, Monivong boulevard, Phnom Penh, Cambodia; E-Mails: pbuchy@pasteur-kh.org (P.B.); mardy@pasteur-kh.org (S.M.)

**Keywords:** magnetic particles, quantum dots, hybridization, DNA, flow cytometry, fluorescence microscopy

## Abstract

In the present study we describe sandwich design hybridization probes consisting of magnetic particles (MP) and quantum dots (QD) with target DNA, and their application in the detection of avian influenza virus (H5N1) sequences. Hybridization of 25-, 40-, and 100-mer target DNA with both probes was analyzed and quantified by flow cytometry and fluorescence microscopy on the scale of single particles. The following steps were used in the assay: (i) target selection by MP probes and (ii) target detection by QD probes. Hybridization efficiency between MP conjugated probes and target DNA hybrids was controlled by a fluorescent dye specific for nucleic acids. Fluorescence was detected by flow cytometry to distinguish differences in oligo sequences as short as 25-mer capturing in target DNA and by gel-electrophoresis in the case of QD probes. This report shows that effective manipulation and control of micro- and nanoparticles in hybridization assays is possible.

## 1. Introduction

Nucleic acid hybridization on solid supports is widely used in biotechnology for the isolation and capture of specific DNA sequences. In particular, many DNA-probe assays utilize oligo-conjugated magnetic particles (MPs) for capturing complementary nucleic acids [1,2]. These MPs are composed of iron oxide nanoparticles embedded in a polymeric matrix and are appropriate for target DNA detection and sample isolation [3–5]. Specific DNA sequences have been detected by hybridization assays using different materials such as biotin-avidin, protein-enzyme, and fluorescent dyes. However, these methods are limited by low signal intensity, rapid photobleaching as well as biosafety problems [6,7]. Fluorescent semiconductor nanocrystals, quantum dots (QDs) possess unique properties and have significant advantages (such as tunable band gap and extraordinary photostability) over the classic organic dyes. Recent advances in nanotechnology have led to a broad range of application fields including using QDs as FRET donors in biosensors, QD-based immunoassays, and in vivo imaging with QDs as a fluorescence marker [8–11].

Our interest in the unique optical properties of QDs has resulted in their combination with microparticles for improved target capturing (solid state separation) and detection with a fast and sensitive diagnostics tool as a final goal.

The synergy of combined MP-based hybridization for target capturing and QD detection techniques will extend the basic application from an analytical device to the detection of complex biological samples or to integrated lab-on-a-chip platforms. This would allow use for fast target DNA detection in point-of-care diagnostics and field analysis. However, interactions between DNA, QD probes, and MP probes are still not well defined and a detailed characterization of the procedure does not exist. A better understanding and optimization of the complex hybridization conditions between functionalized MP/QD probes and nucleic acid targets is necessary for proper assay design, and will provide new insights and possibilities in various biomedical and biotechnological fields.

In this paper, we describe the design of MP-conjugated and QD-based hybridization probes and their application for the detection of the avian influenza virus (H5N1). The hybridization complex comprises three elements in a sandwich manner: (i) MPs with oligo probes on their surface for target capturing, (ii) target DNA derived from a viral sequence, and (iii) QD probes for fluorescence detection. We also addressed the question of whether hybridization efficiency is affected by oligo density on the surface of the MPs and by spacer length. The quality of probe preparation and hybridization reaction was determined by flow cytometry and fluorescence microscopy. To quantify the hybridization efficiency between the target DNA and the labeled probe, the particles-coupled DNA was directly stained with a fluorescent dye specific for dsDNA. For the discrimination of the target strand and non-complementary strand, the labeled MP–target–QD-complexes were analyzed by flow cytometry and microscopy.

## 2. Results and Discussion

**Figure 1.**
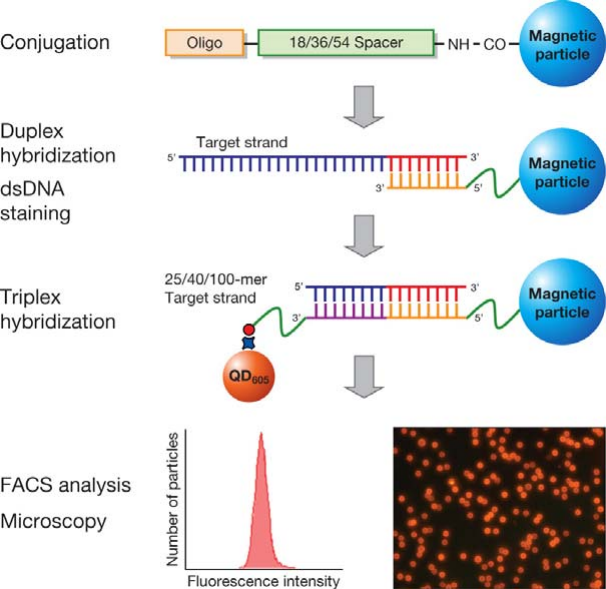
Schematic procedure of the MP and QD-based hybridization assays.

In order to investigate the usage of MPs and QDs as probes in nucleic acid hybridization assays, we designed a MP and QD-based probe assay for the detection of avian influenza H5N1. Figure 1 shows a schematic of the procedure for the hybridization of MP and QD-based probes with the target. Hybridization of 25-, 40-, and 100-mer target DNA with the two probes was performed in 4 steps: (i) conjugation of target specific oligos onto the MP surface, (ii) hybridization of MP probes with the target (of 25-, 40-, and 100-mer), (iii) hybridization of the MP labeled target with a biotinylated oligo, and (iv) detection by QD_605_ streptavidin conjugate.

### 2.1. Determination of Oligo Density on the Surface of the MPs

**Figure 2.**
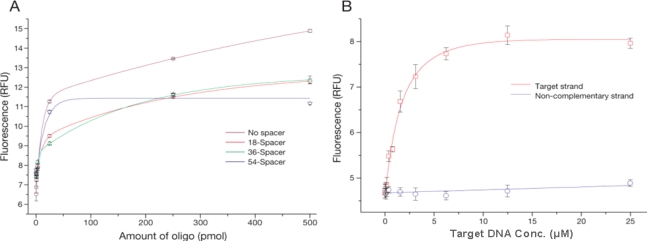
Oligo binding on the surface of MPs and hybridization of the MP probes and target strand. A. Different quantities of oligos with varying lengths of spacers from 0 to 54 chains were conjugated to MPs. After conjugation, they were stained with OliGreen and analyzed by flow cytometry. B. Hybridization of 100-mer target DNA and MP probes. In analogy to A, PicoGreen fluorescence was measured by flow cytometry and plotted as a function of target strand amount.

The oligo density on the surface of MPs might influence the hybridization efficiency; on one hand a very low number of oligos reduces the number of potential binding sites, and on the other a very high density might lead to sterical hindrance. The hybridization efficiency was determined using a calibration curve of known oligo concentration. At the same time, the oligo binding capacity of MPs with spacers of different lengths was characterized to check for their potential impact on the oligo coupling (Figure 2A). When a longer linker was used, a decrease in the amount of oligos bound to the particles was observed (Figure 2A, 54-spacer). This effect is probably due to the formation of secondary structures on the linker, which results in the terminal amino group being unavailable for coupling. In analogy to the coupling process, Steel *et al*. [9] and Peterson *et al*. [10] reported that as the full length of the oligo participates in the hybridization event, high probe density on the particle surface was associated with reduced hybridization efficiency due to steric effects.

The density of conjugated oligos on the MPs for spacer lengths 18, 36 and 54 chains using 500 pmol of oligos was 4.7 × 10^7^, 5.0 × 10^7^, and 4.1 × 10^7^ molecules per MP, respectively, as indicated by indirect measurement of supernatant fluorescence after conjugation. A possible effect of spacer length on target DNA and MP probes hybridization was also investigated. Based on these results (Figure 2A), 500 pmol of oligo with a 36 spacer length was used for MP probes in the following experiments.

### 2.2. Quantitative Analysis of Hybridization of MP Probes and DNA

To verify the hybridization of MP probes and DNA, 1 μL of 100-mer target DNA ranging from 0.05 μM to 25 μM were hybridized on MP probes (5 μL, 2.95 × 10^4^ MP, 5.0 × 10^7^ oligos per MP) in SSC buffer. Several methods such as radioisotope, fluorescence dye labeling, or staining have been developed to reveal the hybridization efficiency [11–13]. However, these methods have limits owing to biosafety problems, photobleaching during sample preparation, and high background noise. We developed a method to discriminate differences in oligo sequences as short as 25-mer capturing to target DNA in the process of hybridization using PicoGreen-based flow cytometry methods. The fluorescence signal from MP probes hybridized with the target strand is very sharp and shifted when compared to MP probes hybridized with non-complementary target strand. A calibration curve was obtained by measuring the fluorescence of individual particles with flow cytometry after staining with a fluorescent dye specifically for nucleic acids (Figure 2B). Fluorescence intensity initially increased rapidly with target concentration (up to 6.4 μM), and started to reach the saturation of the probe hybridization sites at 12.8 μM of 100-mer DNA. In contrast, non-specific binding to non-complementary target DNA was negligible. Based on these results, a quantitative analysis method for the hybridization process was established by flow cytometry for sequences as short as 25-mer, providing valuable information for probe development technology. Our methods show that hybridization reactions for the detection of human pathogens on MPs can progress satisfactorily.

### 2.3. Electrophoretic Mobility Shift Assay

Nolan *et al*. [14] and Ho *et al*. [15] reported on the use of QD labeled oligo probes for hybridization with target DNA. The hybridization efficiency was very low because the QD was a comparatively large particle with an extremely low diffusion coefficient. As a result, we selected streptavidin conjugated QD_605_ and biotinylated oligos as QD probes in this study. The multiple binding sites of a streptavidin-coated QD also increase its chance of binding to the DNA hybrids.

The usage of QDs in hybridization experiments is affected by salt composition, concentration as well as temperature and thus is limited by potential aggregation. Salt concentrations of different reaction buffers were calculated and compared with each other. Occurrence of aggregation after hybridization due to inappropriate reaction conditions was controlled using fluorescence microscopy (data not shown).

**Figure 3.**
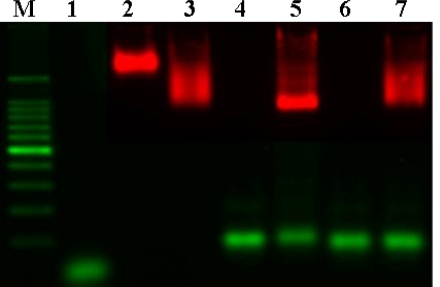
Mobility shift assay of QD probes and DNA hybridization. M: 100 bp ladder, 1: biotinylated 25-mer oligo, 2: QD_605_ streptavidin, 3: QD_605_ streptavidin/oligo, 4: 100-mer target strand, 5: QD_605_ streptavidin/oligo/100-mer target, 6: Non-complementary 100-mer, 7: QD_605_ streptavidin/oligo/non-complementary 100-mer.

Gel electrophoresis was performed to verify the hybridization of QD probes and DNA (Figure 3). Naked QD_605_ streptavidin conjugates, which are surrounded by a negatively charged polymer shell, do not migrate very fast despite their charge (lane 3). Binding of biotinylated oligos (lane 2) to QD_605_ streptavidin conjugates increases the mobility of the particles (lane 4) due to the additional negative charge introduced by the phosphate group on the backbone of DNA. Hybridization of longer target DNA with a higher contribution of negative charges in comparison to the oligos further increases the mobility shift (lane 6). The narrow and clear band indicates the homogenity of the bound population of the target DNA. In contrast, hybridization of the non-complementary target strand (lane 7) and biotinylated oligos/QD_605_ streptavidin conjugate does not reveal a sharp and narrow band, which suggests a heterogeneous population of non-stringent binding (lane 8). From these results, QD probes were shown to be able to hybridize with target DNA by gel electrophoresis.

### 2.4. Fluorescence Microscopy and Flow Cytometry

**Figure 4.**
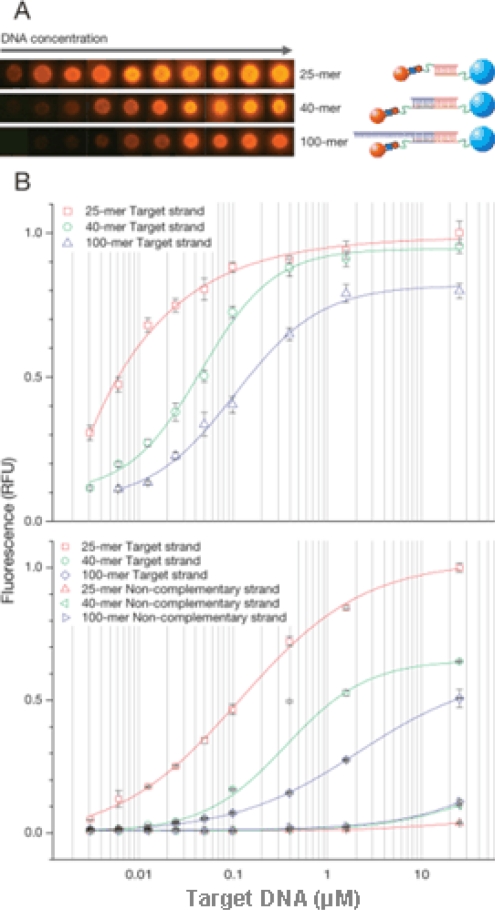
A. Fluorescence images of MPs after hybridization with target DNA of varying length (25-, 40-, 100-mer) and QD probes. B. The hybridization complexes were characterized and quantified by fluorescence microscopy (upper panel) and flow cytometry (bottom panel). Fluorescence intensity is plotted as a function of concentration of the target DNA.

To investigate hybrid complex formation between functionalized MP/QD probes and different nucleic acid target lengths, the hybridization of different length DNA targets (25-, 40-, 100-mer) was compared. Since the thermal stability of the QD is not well-defined [16,17], only low-range room temperatures were investigated. After hybridization, particles were analyzed by fluorescence microscopy and flow cytometry. Uniform fluorescence over particle surface was observed (Figure 4A). In Figure 4B (upper panel), fluorescence intensity increased rapidly with target concentration and reached saturation at about 10 μM of DNA. Hybridization efficiency decreased in case of longer DNA in comparison to shorter DNA. Figure 4B (lower panel) shows the result of flow cytometry analysis after hybridization. The very sharp peak on the count vs. fluorescence intensity histogram shows that the density of DNA is uniform on the surface of MPs. Also shown on Figure 4B is the hybridization of different target DNA lengths (25-, 40-, 100-mer) with the MP and QD probes. In contrast, negligible signals were observed for an excess amount of non-complementary DNA (Figure 4B, lower panel). Target DNA (25, 40, 100-mer) was detected at 3 nM (corresponding to 1 μL of 3 fmol DNA). Further studies are still needed to increase the sensitivity in the application of microdevice in future.

The hybridization efficiency is affected by target length. Longer DNA might affect the biotinylated oligos and QD streptavidin conjugate’s access to the target. With the increasing need for knowledge about interactions of nanoparticles, microparticle-based probes and DNA hybridization, the methods to perform hybridization reactions faster and more accurately are ongoing. Furthermore, optimization of hybridization parameters for QD-probe related DNA hybridization using a microfluidic system is under development. When the target genes are captured on the MP’s surface, genes such as avian influenza virus at low concentrations can be detected. This work shows that it is possible to effectively manipulate and control micro- and nanoparticles in hybridization assays.

## 3. Materials and Methods

### 3.1. Preparation of MP Probes and Characterization of Oligo Density on MPs

Carboxy-functionalized MPs (8.27 μm, 1.554 × 10^8^ mL^−1^, UMC 4N, Bangs Laboratories Inc., USA) were conjugated with amino-functionalized 25-mer oligos (Bioneer, Korea) that were tagged by spacers of different lengths ranging from 18 to 54 carbon chains. Conjugation was performed by carbodiimide (EDC, Sigma-Aldrich, USA) mediated chemistry following a Dynal Biotech protocol (http://www.invitrogen.com).

The target sequences (25-, 40-, 100-mer) were derived from a conserved region of H5N1 avian influenza virus and the non-complementary sequences were taken from the pBluescript SK(+) vector. After conjugation, the MPs were dissolved in 100 μL of PBS buffer (10 mM, pH 7.2, Sigma, USA). The efficiency of the oligo conjugation on the particle surface was determined indirectly by OliGreen (Invitrogen, USA) quantitation of the amount of oligos remaining in the solution phase after immobilization. The total number of particles was measured by flow cytometry adjusting flow rate and elapsed time. Thus, the binding efficiency could be calculated as the ratio of oligos to MPs. To determine the oligo density on the MP surface, 5 μL different amounts of oligos ranging from 0.005 pmol to 100 pmol with spacers of varying lengths were conjugated to 5 μL of MP (8 × 10^5^ particles) and purified using the recommended protocol above. The complexes were stained with OliGreen and analyzed by flow cytometry. Oligo binding capacity of MPs was characterized by flow cytometry.

### 3.2. Hybridization of MP Probes and Target DNA

The hybridization reaction was performed using a slightly modified procedure from Wang *et al.* [18]. For the hybridization of MP probes and the target strand, 5 μL of MP probes were washed once with TTL buffer (100 mM Tris-HCl pH 8.0, 0.1% Tween 20, 1 M LiCl) and resuspended in 5 μL TTL buffer. Subsequently, 1 μL of the target and non-complementary strand ranging from 0.05 μM to 25 μM (2-fold or 4-fold diluted) were added and incubated in 44 μL SSC buffer (750 mmol L^−1^ sodium chloride and 75 mmol L^−1^ sodium citrate) with shaking for 1 h at room temperature. After washing with 100 μL TT buffer (250 mM Tris-HCl, pH 8.0, 0.1% Tween 20), 100 μL TTE buffer (250 mM Tris-HCl, pH 8.0, 0.1% Tween 20, 20 mM Na_2_EDTA), and 100 μL TT buffer, the hybridization complexes were dissolved in PBS and stained with PicoGreen (Invitrogen, USA) and analyzed by flow cytometry. The hybridization efficiency of MP probes and different amounts of target strand was obtained from a calibration curve which was recorded by measuring the average fluorescence intensity of individual particles at known concentrations of target DNA.

### 3.3. Verification of QD Probes

QD_605_–streptavidin conjugates (Q10101 MP, Invitrogen, USA) were used as fluorescent probes for different hybridization complexes: (i) Biotinylated 25-mer oligos with a 36 spacer were designed to be complementary to the oligos attached to the MPs. Biotinylated 15-mer oligos were designed to be adjacent to the MP probes, which are complementary to (ii) 40 and (iii) 100-mer target strands. Gel electrophoresis was used to verify DNA coupling to QD. For confirmation of QD and DNA interaction, QD streptavidin conjugates and biotinylated oligos were mixed together at the ratio of 1:10 and incubated in SSC buffer for 1 h at room temperature. After that, the target strand was added in equimolar amounts of QD streptavidin conjugates. The QD streptavidin conjugates/biotinylated oligos (QD probes) and the target strand hybrid were diluted in loading buffer and separated by gel electrophoresis (2% agarose in 0.5× tris-borate-EDTA buffer at 10 V/cm). The gel was illuminated and analyzed with an RAS 3,000 Image Analyzer (Fuji Film, Japan).

### 3.4. Hybridization of Target DNA, MP Probes, and QD Probes

The MP probes (5 μL) were prepared and hybridized with the target ranging from 0.003 μM to 25 μM (2-fold or 4-fold diluted) as described above. The hybridization complexes were then consecutively washed with 100 μL TT buffer, 100 μL TTE buffer, and 100 μL TT buffer. After washing, they were resuspended in the 50 μL SSC buffer containing biotinylated oligos and incubated with shaking at room temperature for 1 h. The molar ratio of biotinylated oligos and target strand was 1:1. After hybridization, particles were washed twice with TT buffer, resuspended in TTL buffer containing 1 μL of QD_605_ streptavidin conjugates (1 μM) and incubated at room temperature for 1 h. After reaction, particles were washed one more time with TT buffer and dissolved in PBS buffer for flow cytometry and fluorescence microscopy analysis.

### 3.5. Data Acquisition and Analysis

Fluorescence images of magnetic particles hybridized with different length DNA targets were acquired using a conventional widefield microscope (IX-71, Olympus, Japan) equipped with a CCD camera (ProgRes C10plus, JENOPTIK Laser, Optik, Systeme, Germany). Ten fluorescent particles each were selected randomly for the acquisition; their fluorescence intensity was measured using the ImageJ software (National Institutes of Health, USA) and plotted as a function of the amount of DNA. At the same time, the fluorescence of the complexes was also analyzed by flow cytometry (Canto II, Becton-Dickinson, USA) using excitation at 485 nm and emission detection at 530 nm (OliGreen and PicoGreen) or 585 nm (QD_605_). Forward and side scattering were used for the separation of single particles based on size discrimination. The mean fluorescence intensity of the particles was plotted as a function of amount of the target DNA. Typically, 10,000 events per MP were recorded to analyze the data by the BD FACSDiVa software (Becton-Dickinson, USA).

## 4. Conclusions

In this report, the design and performance of the MP and QD probe-based method for identifying and quantifying avian influenza virus sequences was described, and detection methods for the hybridization between micro- and nanospheres with target DNA were established. We determined the oligo density on the surface of MPs and investigated spacer effects on the hybridization between MP probes and target DNA. The accessibility and affinity of the oligos to the target DNA were detected by flow cytometry. We emphasize that sequence differences as short as 25-mer in hybridization can be detected by flow cytometry. The hybridization patterns of single particles were visualized in a sandwich hybridization assay, and hybridization efficiency as a function of target strand length was presented, showing that steric hindrance prevented QD probes accessing the target strand. The established system will be used to miniaturized analytical devices for fast detection of target DNA based on MP and QD probes.
